# Limb lengthening in congenital posteromedial bow of the tibia

**DOI:** 10.1007/s11751-012-0145-4

**Published:** 2012-10-16

**Authors:** S. D. Kaufman, J. A. Fagg, S. Jones, M. J. Bell, M. Saleh, J. A. Fernandes

**Affiliations:** 1 Killick Health Centre, London, UK; 2 Department of Paediatric Orthopaedics and Trauma Surgery, Paediatric Limb Reconstruction Service, Sheffield Children’s Hospital, Western Bank, Sheffield, S10 1UY UK; 3 Norwich Hospital, Norwich, UK

**Keywords:** Limb lengthening, Posteromedial bow, Deformity correction

## Abstract

Congenital posteromedial bowing of the tibia (PMBT) is a rare condition affecting one lower limb. The bowing of the tibia usually resolves; however, there is associated limb length discrepancy (LLD), which often persists and can cause functional deficits. Advances in limb lengthening techniques allow this issue to be addressed, often with concomitant angular deformity correction. This study examined eleven patients who have had limb lengthening procedures with mean pre-operative LLD of 3.7 cm (range 1.5–5 cm), mean increase in length was 3.9 cm (range 1.5–5.8 cm), and mean LLD at last follow-up was less than 0.6 cm (range 0–2.0 cm). The main complications were minor or moderate grades, such as pin site infection. Greater LLD was found than previously reported, and we believe that the tertiary referrals were those of a severe form of PMBT. The authors conclude that in view of deformity with discrepancy, in select cases, correction and lengthening would be an option rather than only contralateral epiphysiodesis.

## Introduction

Congenital PMBT is a rare condition. It was first fully described in 1949 by Heyman and Herndon [1]. It is also known as kyphoscoliosis of the tibia or posterior angulation; since 1949, there have been very few papers published about the condition.

Posteromedial bowing of the tibia usually presents at birth with a bowed, shortened leg. The angulation of the tibia and fibula is in the medial and posterior direction—with equal angle of bowing (varying from 25° to 70°) usually in the middle or distal third of the shaft [2]. The anomaly is unilateral, not associated with any other abnormality [3], accompanied by an initial calcaneovalgus deformity of the foot and decrease in ankle motion that does not improve with age. The deformity can vary in severity; the most severe is when the dorsum of the foot is in contact with the anterolateral aspect of the lower leg. The amount of bowing has been reported to be unrelated to the associated shortening [3, 4], although a more recent review showed the degree of tibial shortening to be related to the degree of medial bowing, but not posterior bowing [5].

The aetiology of the deformity is unknown; the prognosis is good: Normally, there is a spontaneous, although incomplete, correction of the bowing within the first 4 years of life. The shortening increases with age [2, 6] to a length deficit of 4**–**7 cm at maturity [4] and is the most serious sequel. It may be serious enough (greater than 2 cm) to require limb length equalisation [7]. The proportionate difference in leg lengths remains stable throughout childhood, thus allowing a prediction of leg length discrepancy to be accurate within 2 mm using estimates made from as early as 3 years old [4].

The treatments outlined in the literature include conservative treatment with serial splints or casts or surgery. Surgical intervention initially was only contralateral epiphysiodesis (growth arrest) [3] and then included limb lengthening and contralateral limb shortening [4]. Pappas has published the largest series with 33 patients, 30 of whom had contralateral epiphysiodesis and only one of whom had limb lengthening, all patients had a good outcome. We intend to study our experience in limb length equalisation using distraction osteogenesis in this select group referred to our tertiary centre.

## Methods

We identified 17 patients diagnosed with congenital PMBT, from the Paediatric Limb Reconstruction Service (PLRS) database between 1989 and 2002, 11 of which underwent and completed leg lengthening procedures during this time. The PLRS is a tertiary referral service that almost certainly receives the more severe or difficult to treat patients with PMBT, which in our experience is a clear subset of patients with the condition [5]. The patient’s year of birth spanned 1979**–**1992.

The inclusion criteria were a diagnosis of congenital posteromedial bow and leg lengthening. In some patients, the deformity correction was mostly angular, but they have been included if they had any concomitant lengthening. This explains why one patient had only 15 mm of lengthening. This is a retrospective study looking at clinical notes, dictated letters, operation notes and radiographs. Data were extracted from the notes and radiographs and used to calculate the mean LLD, ankle range of movement and deformity both pre-operatively and post-operatively. The type(s) of operation, length gained and duration of lengthening were noted. Specific attention was paid to complications. The bone healing index was calculated by dividing the number of days that the patient had the frame on by the number of centimetres gained in the length of the bone.

The two correction devices used were the Limb Reconstruction System (LRS), a monolateral device, manufactured by Orthofix and the Ilizarov frame, a circular fixator, manufactured by Smith and Nephew. All patients had intensive physiotherapy during and after surgery until good range of movement was achieved.

The radiographs that were selected were as follows: the standard standing mechanical axis pre-operatively and at last follow-up and the lateral view of the tibia pre-operatively and at last follow-up. The magnitude of the oblique plane angle of deformity was calculated using the centre of rotation of angulation (CORA) method [8]. Leg length discrepancy was measured from full-length radiographs of both limbs and clinical assessment.

## Results

**Fig. 1:**
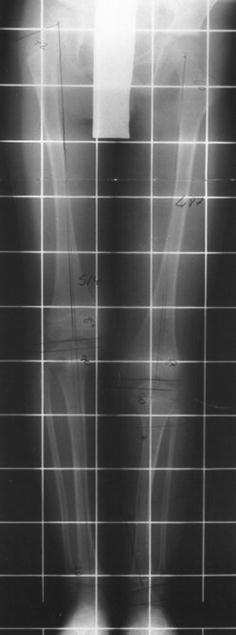
Radiograph of pre-operative mechanical axis

**Fig. 2:**
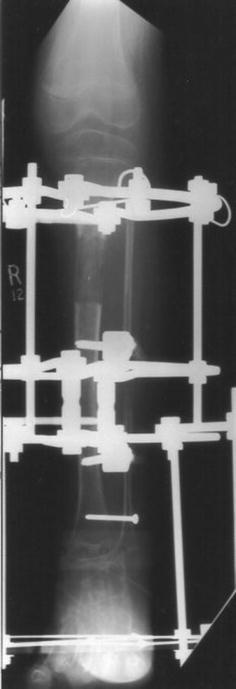
Radiograph AP view during lengthening

**Fig. 3:**
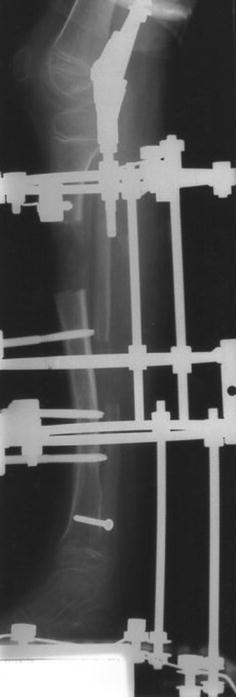
Radiograph lateral view during lengthening

**Fig. 4:**
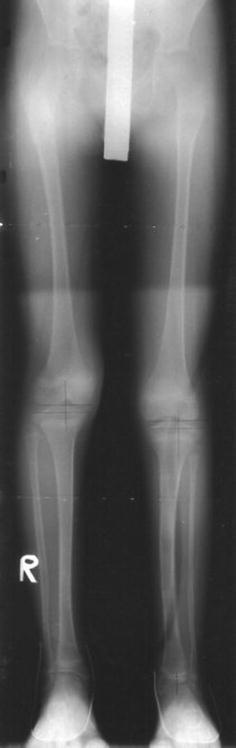
Radiograph of follow-up mechanical axis view

The eleven patients referred to our tertiary referral centre were six boys and five girls. The mean age at index operation was 11 years (3.2**–**17.4 years). The index frame used for four of the patients was the Ilizarov frame, with a monolateral device for the remaining seven patients (see Figs. 1, 2, 3, 4). Patients treated with the Ilizarov frame had gradual angular correction as well as lengthening, while those treated with a monolateral device had acute angular correction at frame application, followed by lengthening. Follow-up from the date of removal of the index frame was 4.9 years (1.8–9.5 years). The mean age at final follow-up was 15.5 years (6.3–21.1 years).

The mean pre-operative LLD was 3.7 cm (range 1.5**–**5 cm). Two of the patients treated with Ilizarov frames had pre-operative angular deformity within one plane (30° posterior angulation and 15° medial angulation). The other two had oblique angular deformities (39° and 33°).

The patients exhibited various degrees of ankle restriction from 0° dorsiflexion and 10° plantar flexion (in different patients) to normal ankle movements. Those with decreased movement were usually restricted dorsiflexion; six patients had less than 5° of dorsiflexion.

The mean length gained in index operation was 3.9 cm (range 1.5–5.8 cm). The mean frame time was 247 days (range 87–586 days), giving a mean healing index of 66 days per cm (range 30–169 days/cm). Mean leg length discrepancy at final follow-up was less than 0.6 cm (range 0–2 cm).

The operative results are presented in Table 1.

**Table 1:** Pre-operative and final LLD, bone healing index and frame type

Patient number	Pre-operative LLD (cm)	Bone healing index (days/cm)	Length gained (cm)	Frame used	Final follow-up LLD (cm)
1	4.5	38.7	4.5	LRS	0.6
2	3.0	35.3	4.0	Ilizarov	1.5
3	3.5	67.2	3.2	LRS	0.6
4	4.3	123.6	4.5	Ilizarov	0.3
5	3.0	30.0	3.0	Ilizarov	0.0
6	5.0	68.8	5.0	LRS	0.6
7	5.0	34.8	5.8	LRS	2.0
8	1.5	169.3	1.5	LRS	0.0
9	3.5	40.9	3.4	Ilizarov	0.0
10	3.0	81.2	2.5	LRS	0.8
11	4.3	32.3	5.2	LRS	0.0

Post-operatively, the range of ankle movement was unaltered.

The complications are listed in Table 2; they were classified by Saleh and Scott [10], into mild, moderate and severe type I or II (see Table 3). Mild complications were considered to be of no long-term functional or anatomical significance and included pin site infections.

**Table 2:** Operative complications and frequency

Complications	Frequency
Pin site infection responding to oral therapy	4
Pin site infection responding to IV therapy	4
Operation to stabilise fibula	4
Operation to change device	2
Bone graft	1
Mal-alignment requiring further operation(s)	2

**Table 3:** Complications as classified by Saleh et al. [10]

Mild	Moderate	Severe type I	Severe type II
8	7	2	0

Moderate complications required general anaesthesia but are of no long-term significance, for example, insertion of *K*-wires. Severe type I complications cause a significant functional or anatomical problem that improves spontaneously or is correctable by operation, including angular deformities of greater than 10° in the tibia. Severe type II complications are irremediable by conventional treatment and include permanent nerve damage (as shown in Table 3).

Two patients reported anterior knee pain and one patient ankle pain, neither had any observable mechanical cause nor functional complications. The mean number of complications per completed operation (i.e. frame removed) is 17/11. However, most of the complications were mild or moderate, with no severe type II complications. We can postulate that most were of no long-term significance. We did not count the need for further angular corrections or contralateral epiphysiodesis as complications of the limb lengthening.

Three of the patients treated with the Ilizarov frame had no angular deformity following index frame removal, and one had a residual 10° valgus deformity but did not require further angular correction. Three of the patients treated with a monolateral device required further operations for residual angular correction (ranging from 10° uniplanar deformity to 37° oblique deformity), undergoing six procedures, with five Ilizarov frames and one monolateral device. Four of the patients initially treated with monolateral devices had successful angular correction and did not require further surgery. The mean residual deformity in any plane was 1°**–**2°.

Two patients underwent contralateral epiphysiodeses, performed at age 15 years and 16 years 1 month, respectively. Both epiphysiodeses were at the contralateral distal femur and proximal tibia and fibula to keep the knees in alignment, as the LLD decreased and equalisation was achieved. Both had finished limb lengthening, and one gained 1.2 cm and the other 2.9 cm of equalisation. Periosteal stripping was performed once, and the benefit was 0.5 cm; it is now thought to have little long-term benefit [9].

## Discussion

Congenital PMBT is a rare condition. In our tertiary referral centre, we identified 17 referrals in 13 years. The largest series is reported by Pappas [4]. He had 33 patients, of whom 30 had or were due to have contralateral epiphysiodesis and one had a lengthening operation. Johari et al. and Shah et al. [5, 11] have more recently published series of 31 patients (six of whom had lengthening procedures) and 20 patients (two of whom had lengthening procedures), respectively.

The aetiology of the deformity is unknown and controversial [12]. It appears that the primary defect in the disorder may have occurred in the embryological development of the lower leg [4, 6]. Other theories have included intrauterine fracture, abnormal foetal position or restriction of growth from soft-tissue contractures or amnion rupture [13]. Weight is given to the embryological theory by X-ray findings and the fact that the muscles and subcutaneous tissue are affected. This would imply the initial insult might have arisen in the stage of development after formation of the cartilaginous model and during primary ossification [14].

The subsequent abnormalities seen would then be a consequence of a developmental abnormality—this is further strengthened by the inherent growth deficit that is permanent and present throughout childhood—and the persistent internal tibial torsion [14].

Other associated clinical findings in children with congenital PMBT include the following: underdevelopment of the calf muscles, decreased length and width of the foot [7] and, as stated previously, a decrease in ankle movement, which does not improve. In this series, there was decreased pre-operative arc of ankle movement, which was unchanged post-operatively. We found one report in the literature of a case of bilateral congenital PMBT [5].

The medial and fibula bows are the components that are longer lasting [4]. The other abnormal X-ray findings, a thickened cortex on the concavity of the curve on both tibia and fibula and characteristically undifferentiated intramedullary canal [4] resolve with age in the tibiae, however, may persist in the fibulae, but to a lesser extent. Irregularities may appear in the fibula, in some adolescents [4]. In our patients, similar findings were observed on X-rays, and there were no additional irregularities on fibulae appearing in adolescents.

Unlike other types of congenital angular deformities of the tibia and fibula—including anterolateral and anteromedial bowing—there is no increased tendency to fracture, pseudoarthrosis [7] or neurofibromatosis [15]. None of the patients in our series demonstrated these features. Congenital PMBT is the rarest of the three. Inhibition of growth at the distal tibial physis appears to be the primary factor implicated in the shortening [4] and thus causes shortening throughout growth. Bone healing is not a problem after a corrective osteotomy as there is no underlying bone disorder affecting healing [16]; in this series, one patient required a bone graft for a deficient anterior cortex. A patient who had been operated on previously and required a bone graft and plating was referred to our unit and was further operated on successfully, indicating that the bone was healthy.

There is a huge range of deformity, from no perceivable lasting deformity or LLD to that of one patient, who had about 8 cm LLD (5 cm lengthening and then epiphysiodesis when LLD reached 3.2 cm) and required numerous operations. Previously, the greatest LLD in the literature was 7 cm, and the smallest was 3 centimetres at maturity [17]; there is no record of resolution without LLD. Pappas had a mean LLD at maturity of 4.1 cm (range 3.3**–**6.9 cm) [4] which is comparable with our pre-operative mean; however, our mean was not at skeletal maturity, perhaps reflecting that this is a tertiary referral centre, and the cases were possibly from the more severe end of the spectrum.

The patient that was referred following a previous operation had a LLD of 8.8 cm prior to that operation. She then required further corrective surgery where she gained 4.5 cm. The initial operations could have altered the growth potential, especially as there were complications requiring a bone graft; however, the discrepancy was 8.8 cm and therefore greater than that previously published.

All patients achieved the target aim for length, and the mean final follow-up LLD was 0.6 cm.

The mean healing index of 66 days per cm is relatively large; however, the distribution is positively skewed due to the two patients (see Table 1, patients 4 and 8 who had delayed regenerate formation). At the time that these two were operated on, we had a requirement for all four cortices to have consolidated prior to frame removal. It is now generally accepted that three cortices are sufficient, and hence, their healing indices would be reduced had this been applied. All of the children also had an element of angular deformity correction in addition to lengthening. Without those two patients, the mean healing index would have been 48 days per cm, which is comparable with other studies [10, 18, 19]. The bone healing index with each type of frame (LRS and Ilizarov) was analysed for any statistically significant difference using the Mann–Whitney *U* test. The mean bone healing index with the Ilizarov frame was 57.5 and 70.3 days/cm for the LRS. This difference was not statistically significant (0.648, significance level 0.05). After excluding the two outliers (one in each group), the difference comes closer to significance (0.381) but is still not statistically significant.

Historically, early osteotomy was advocated to hasten the correction of the bow [4, 6, 14]. Pappas performed four osteotomies and showed that the eventual LLD was unaffected [4]. In this series, all the patients had deformity correction, seven acutely and four using the Ilizarov ring fixator.

The six additional corrective operations were performed on three patients, who had mal-alignment, and had initially been operated on using a monolateral device. Two of these were operated on early in the series, during the learning curve. There were four operations using monolateral devices that were successful—they did not cause mal-alignment. Three of the seven patients treated with the monolateral fixator had significant residual angular deformity requiring further correction. None of the four patients treated with the circular frame had significant residual deformity. The complication rate and distribution were comparable with other lengthening procedures [10, 18]. There were no soft-tissue complications that restricted function and no deep infections at final follow-up.

As previously stated, Pappas treated one case with lengthening. The patient required a subsequent bone graft and was only partially corrected [4]. Shah et al. [5] treated two patients with lengthening. One had residual LLD of 13 % tibial shortening at 12-year age, and the other 15 % (although it is not specified whether this is pre- or post-operative). Johari et al. [11] presented six patients treated with leg lengthening, with an average pre-operative LLD of 3.93 cm (3.2**–**5.4 cm), comparable with our own. Mean residual LLD was 0.8 cm (0**–**2.5 cm), also comparable with our own.

This is the largest series of patients to have this condition treated with leg lengthening procedures, and although there have been complications which we could largely attribute to our initial learning curve, the eventual outcome has been good, with the mean eventual LLD being less than 0.6 cm at the last follow-up. Only two patients had a LLD over 0.6 cm, one at 1.5 cm and the other at 2 cm, who are happy with shoe raises.

## Limitations

This is a retrospective review and is, therefore, limited by the heterogeneity of the available data and follow-up. With further detailed documentation, it may be possible to clarify many more issues, such as the ideal timing of surgery and techniques, even with the small numbers available. Also, the fact that we are a tertiary referral centre is likely to mean that the cases presenting to us are at the more severe end of the spectrum or are showing less tendency to correct themselves.

## Conclusions

Contralateral epiphysiodesis is a simpler option for limb length equalisation; however, given that deformity correction and lengthening are successful and will not reduce eventual height, it should be considered as an option, especially in severe cases. The circular fixator has given us better results than the monolateral device with regard to achieving and maintaining satisfactory alignment. Partly, this may be because the deformity could be dealt with in the oblique plane and a more stable construct for correction and lengthening can be achieved. It may, however, also be related to the fact that the patients treated with the monolateral device had acute angular correction, whereas the patients treated with the circular frame had gradual correction. There was no statistically significant difference in the bone healing index between the two devices.

This is the largest series of lengthening and correction for this condition. We have found that successful simultaneous deformity correction and lengthening for this condition is possible.
